# Sarcomatoid Mesothelioma Revealed After Treatment for Tuberculous Pleurisy: A Case Report

**DOI:** 10.1002/rcr2.70734

**Published:** 2026-09-01

**Authors:** Daiki Nagayama, Keiki Yokoo, Yousuke Yabushita, Motoki Sekikawa, Masaru Abe, Hiroaki Kato, Ayumi Takayanagi, Bunya Takahashi, Marie Kato‐Shinomiya, Satoshi Ota, Gen Yamada, Kenzo Hiroshima, Hirofumi Chiba

**Affiliations:** ^1^ Department of Respiratory Medicine Teine Keijinkai Hospital Sapporo Japan; ^2^ Department of Thoracic Surgery Teine Keijinkai Hospital Sapporo Japan; ^3^ Department of Diagnostic Radiology Teine Keijinkai Hospital Sapporo Japan; ^4^ Department of Pathology Teine Keijinkai Hospital Sapporo Japan; ^5^ Department of Pathology Tokyo Women's Medical University Yachiyo Medical Center Chiba Japan; ^6^ Department of Respiratory Medicine and Allergology Sapporo Medical University School of Medicine Sapporo Japan

**Keywords:** fluorescence in situ hybridization (FISH), pleural biopsy, sarcomatoid mesothelioma, tuberculous pleurisy

## Abstract

Differentiating tuberculous (TB) pleurisy from malignant pleural mesothelioma is challenging due to similar clinical presentations. We report an 83‐year‐old man with sarcomatoid mesothelioma initially treated for TB pleurisy. He presented with lymphocyte‐predominant exudative pleural effusion and a positive Interferon‐Gamma Release Assay. Thoracoscopy revealed purulent effusion, but biopsies were non‐diagnostic. Despite empiric anti‐tuberculosis therapy, the effusion persisted. A subsequent surgical biopsy showed fibrosis and scattered spindle cells. However, 2 months later, a rapidly growing chest wall mass with bone invasion appeared. A CT‐guided percutaneous biopsy showed proliferation of atypical spindle cells and fluorescence in situ hybridization (FISH) analysis allowed for the diagnosis of sarcomatoid mesothelioma. This case highlights the aggressive nature of sarcomatoid mesothelioma and its diagnostic difficulty. For treatment‐refractory pleurisy, clinicians must maintain high suspicion, ensuring vigilant follow‐up and prompt repeat biopsies despite negative initial results, while consulting pathologists to consider additional FISH testing.

## Graphical abstract

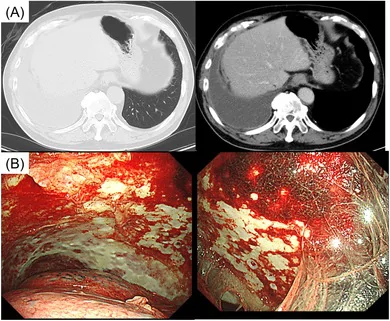

## 1 Introduction

Distinguishing tuberculous (TB) pleurisy from pleural mesothelioma (MPM) is often difficult due to overlapping clinical presentations, such as lymphocyte‐predominant exudative effusions. We describe a case of sarcomatoid mesothelioma in an elderly male initially managed as TB pleurisy. Notably, a chest wall mass appeared rapidly, even after a surgical biopsy revealed non‐specific findings predominantly characterised by fibrosis. This case demonstrates that vigilant follow‐up, repeated biopsies, and multidisciplinary discussion can overcome complicated diagnostic challenges.

## 2 Case Report

**FIGURE 1:**
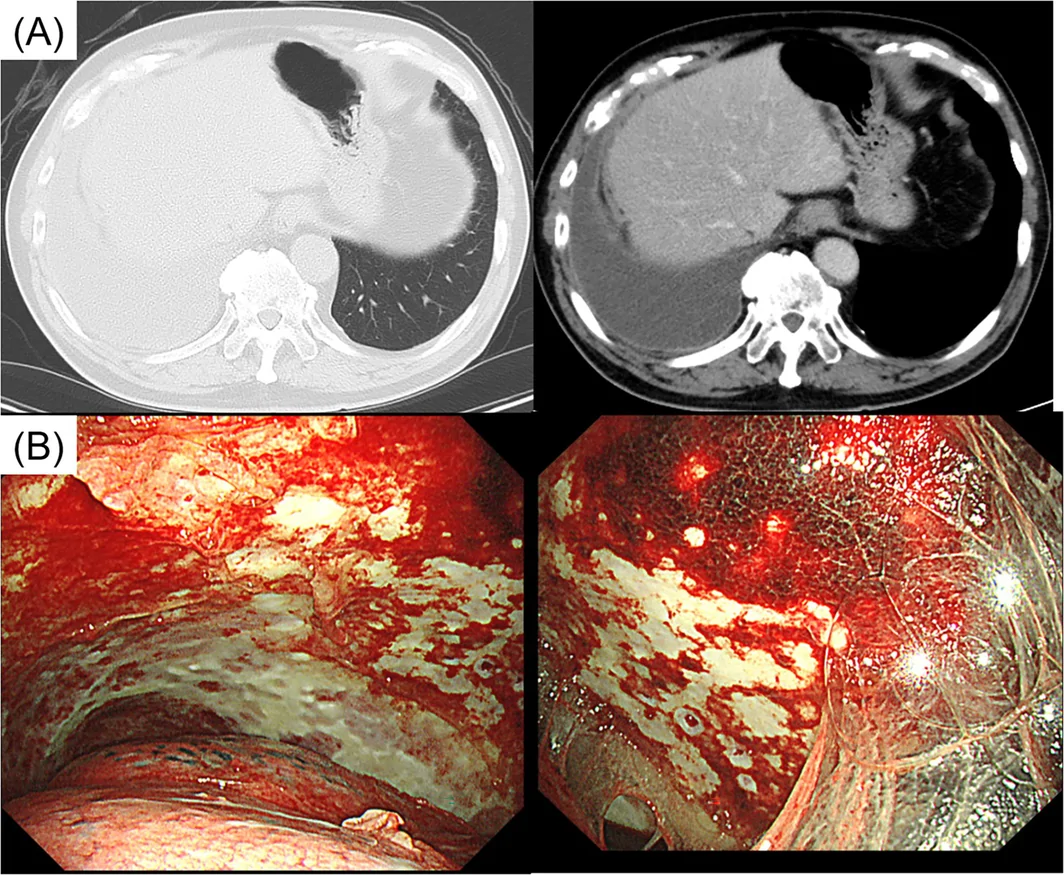
(A) Chest Computed Tomography (CT) at the initial presentation (November 2024) showing massive right pleural effusion. (B) Thoracoscopic view at the referring hospital showing purulent effusion and fibrin deposition in the pleural cavity.

**FIGURE 2:**
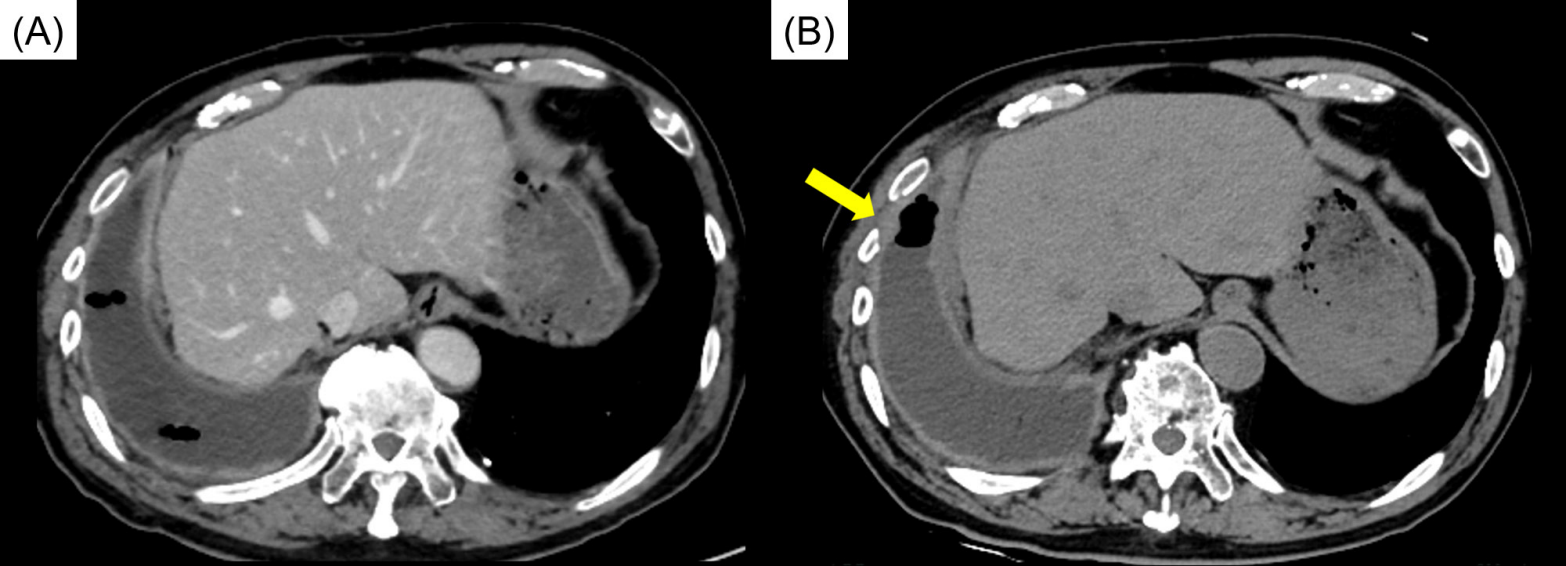
(A) Chest CT before surgical biopsy (June 2025) showing diffuse right pleural effusion and pleural thickening, but no obvious mass formation. (B) Chest CT performed 2 months after the surgical biopsy (August 2025), revealing a new mass with bone invasion in the right 8th rib (arrow).

**FIGURE 3:**
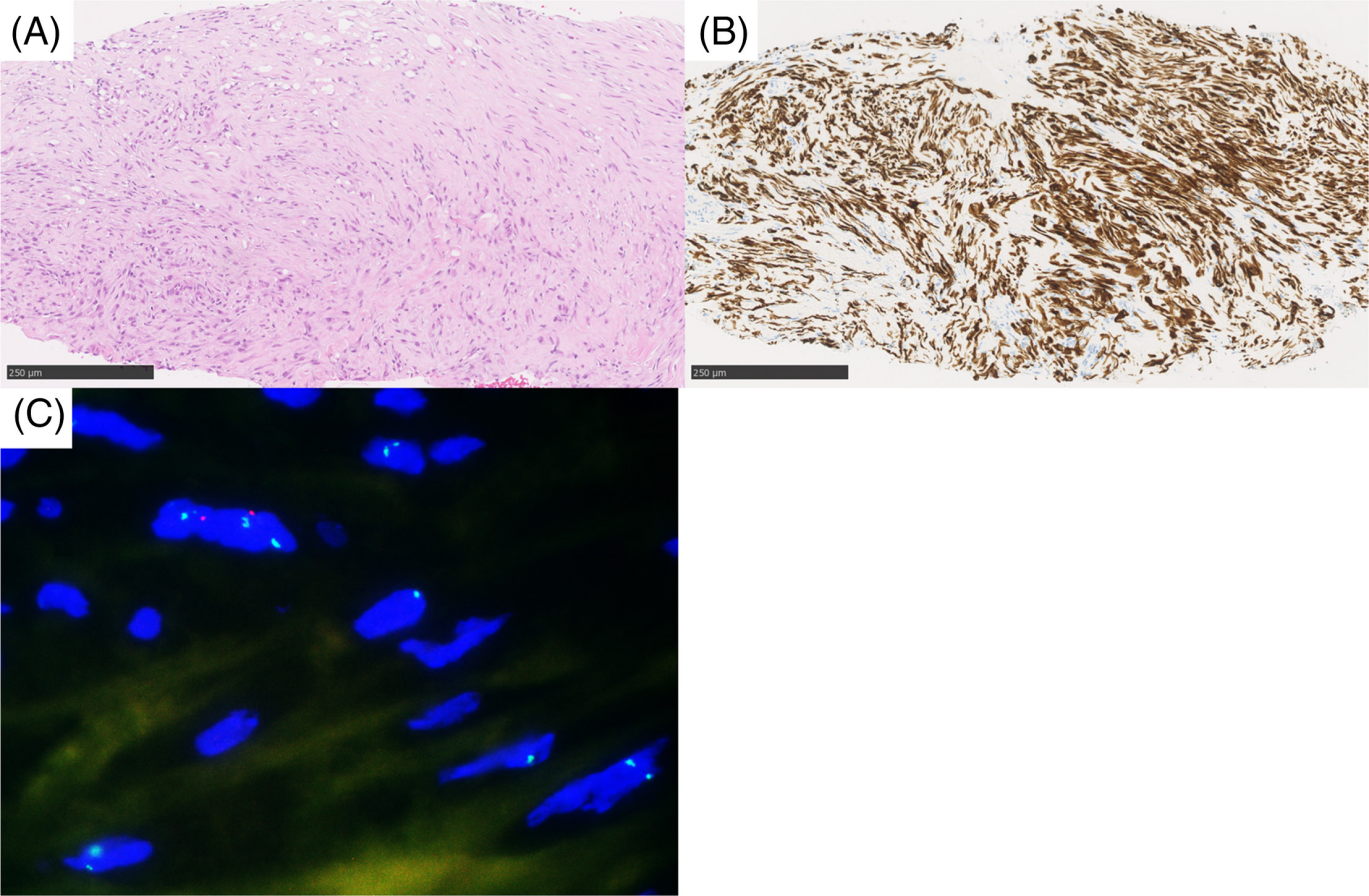
Histopathological and FISH findings of the percutaneous pleural needle biopsy: (A) Proliferation of atypical spindle cells with nuclear enlargement and irregularity, accompanied by fibrosis (Haematoxylin and Eosin stain). (B) Immunohistochemical staining showing that the spindle cells are positive for CAM5.2. (C) FISH showing homozygous deletion (loss of both red signals) of CDKN2A (p16). Arrows indicate non‐tumour cells (two red signals and three green signals).

An 83‐year‐old man with a history of surgically treated colon cancer (pStage IIIA) presented to a referring hospital in November 2024 with right pleural effusion (Figure 1A). He had a smoking history of 10 pack‐years and an occupational history involving administrative work at construction sites. The pleural fluid was a lymphocyte‐predominant exudate. Cytology was negative for malignancy. Although the Interferon‐Gamma Release Assay (IGRA) was positive, pleural fluid adenosine deaminase (ADA) levels were only mildly elevated at 40.5 U/L. Thoracoscopy performed under local anaesthesia revealed purulent effusion (Figure 1B), but an adequate pleural biopsy could not be obtained due to inflammation. Clinically suspecting TB pleurisy, empiric treatment with anti‐tuberculosis drugs (isoniazid, rifampicin, ethambutol, pyrazinamide) was initiated in January 2025. However, the pleural effusion persisted, and PET‐CT showed no abnormal accumulation suggestive of malignancy. This patient was referred to our hospital in April 2025. Because the pleural effusion had not resolved despite 4 months of antituberculosis therapy, a thoracoscopic biopsy was performed at the site of pleural thickening identified on a preoperative CT scan (Figure 2A). Histopathology revealed fibrous pleural thickening with no evidence of granulomatous inflammation or acid‐fast bacilli on Ziehl–Neelsen staining; tissue cultures for mycobacteria and general bacteria were negative. In August 2025, a follow‐up CT scan performed 2 months postoperatively revealed a new mass with bone invasion in the posterior right 8th rib (Figure 2B). The patient reported pain in the area. A CT‐guided percutaneous biopsy of the mass was performed. Histopathologically, the observed fibrosis and proliferation of atypical spindle cells made it difficult to distinguish between fibrosing pleuritis and sarcomatoid mesothelioma (Figure 3A). Immunohistochemically, the spindle cells were diffusely positive for CAM5.2 (Figure 3B). To clarify this diagnosis, fluorescence in situ hybridization (FISH) analysis was performed, showing a homozygous deletion of *CDKN2A* (p16), which confirmed the diagnosis of sarcomatoid mesothelioma (Figure 3C). Two months after the definitive diagnosis, the patient died under best supportive care. Clinically, this represented an aggressive course consistent with mesothelioma.

## 3 Discussion

This case underscores two major clinical challenges: the difficulty of diagnosing TB pleurisy and the crucial role of FISH when clinical courses contradict histological findings.

First, diagnosing TB pleurisy is challenging. The sensitivity of pleural fluid smears for acid‐fast bacilli is less than 10%, and cultures are positive in less than 30% of cases [1]. While ADA is a useful marker, its specificity is limited. In this case, the positive IGRA and lymphocyte‐predominant effusion strongly supported the initial clinical diagnosis of TB pleurisy. Sarcomatoid mesothelioma is associated with a poorer prognosis and more rapid progression compared to other histological subtypes [2]. Furthermore, desmoplastic mesothelioma, a subtype of sarcomatoid mesothelioma, is known to present with early lesions that are pathologically indistinguishable from benign fibrous pleuritis [3]. Previous studies report that 5%–15% of patients diagnosed with ‘non‐specific pleuritis’ after invasive biopsy are eventually diagnosed with malignancy, particularly mesothelioma [4, 5, 6]. This suggests that the initial presentation in our case, characterized solely by pleural effusion, likely represented an ‘occult’ phase during which malignant findings were radiologically and pathologically undetectable. This may also explain the negative FDG‐PET findings, as the tumour burden or metabolic activity at that stage may have been insufficient to produce detectable FDG uptake. Therefore, in cases of presumed TB pleurisy showing poor response to empiric therapy, clinicians must promptly perform or repeat pleural biopsies to rule out malignant mesothelioma, rather than persisting with ineffective treatment. The British Thoracic Society (BTS) guidelines recommend a follow‐up of approximately 12 months for cases with non‐specific biopsy results [7].

**FIGURE 4:**
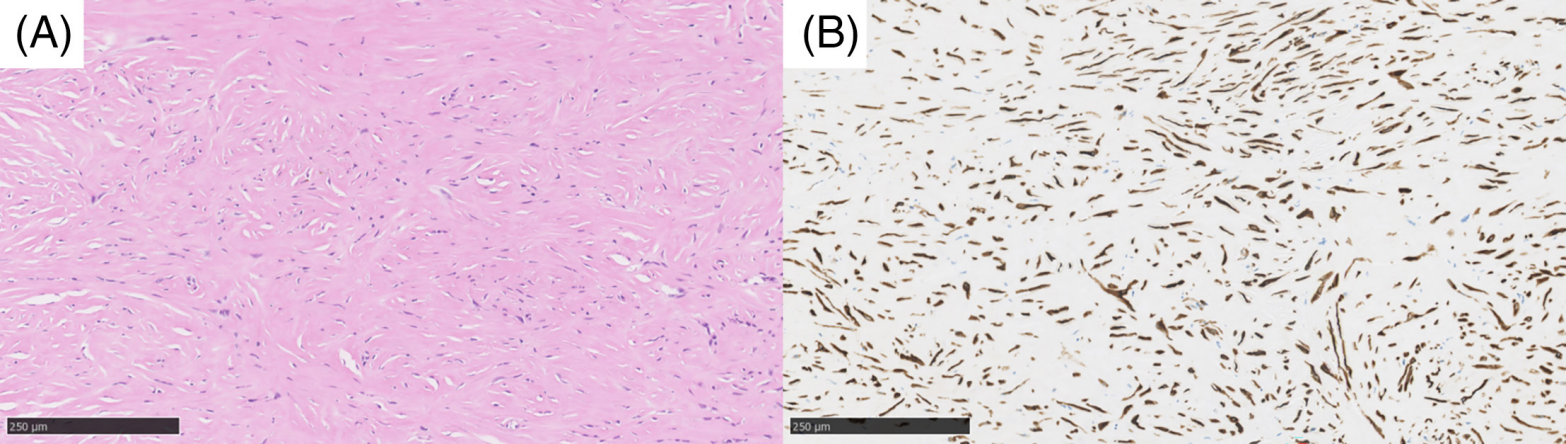
Retrospective histological findings of surgical pleural biopsy. (A) Scattered atypical spindle cells within the predominantly fibrotic pleural thickening (Haematoxylin and Eosin stain). (B) Immunohistochemical staining showing that the spindle cells are positive for CAM5.2.

Second, differentiating sarcomatoid mesothelioma from benign fibrosing pleuritis using routine histology alone remains extremely difficult. In our patient, the findings of the surgical biopsy did not rule out tuberculous pleuritis, but the macroscopic mass formed within just 2 months was consistent with the tumour's rapid growth rate. Retrospective immunohistochemical staining showed that the scattered spindle cells are positive for CAM5.2, similar to the findings of the CT‐guided percutaneous biopsy (Figure 4A,B). Furthermore, retrospective FISH analysis also detected a homozygous deletion of *CDKN2A* (p16) in this sample (data not shown), suggesting that it represented a continuous neoplastic process. When a contradictory clinical course occurs—such as rapid tumour growth despite non‐specific biopsy results—performing FISH to detect the homozygous deletion of CDKN2A (p16) is desirable to establish a definitive diagnosis, as demonstrated in our case. FISH is useful in the diagnosis of sarcomatoid mesothelioma because homozygous deletion of CDKN2A (p16) is frequently observed in sarcomatoid mesothelioma but is absent in benign fibrosing pleuritis. Therefore, assessment of CDKN2A (p16) homozygous deletion by FISH is valuable for distinguishing sarcomatoid mesothelioma from benign fibrosing pleuritis [8]. However, the limited prevalence and availability of FISH testing in general clinical practice remain a significant challenge. The lack of widespread access to this tool could delay the diagnosis of mesothelioma, posing a significant challenge in clinical settings. This case highlights the need for prompt repeated biopsies for patients with a poor response to treatment for presumed TB pleurisy, and underscores the urgent need to expand access to FISH testing to resolve diagnostic challenges.

## Author Contributions

D.N. and K.Y. drafted and revised the manuscript. D.N., K.Y., Y.Y., M.S., M.A., H.K., A.T., B.T., M.K.‐S., S.O., G.Y., and K.H. were involved in the diagnosis and treatment and approved the manuscript. H.C. reviewed the manuscript. All authors approved the final version.

## Ethics Statement

This case report was approved by the Ethics Committee of the Institutional Review Board of Teine Keijinkai Hospital (permit no.: 3‐026007‐00). Written informed consent was obtained from the patient for publication of this case report and any accompanying images. This study was performed in accordance with the Declaration of Helsinki. The CARE Checklist has been completed by the authors for this case report, and provided to the Journal at submission.

## Consent

The authors declare that written informed consent was obtained for the publication of this manuscript and accompanying images using the consent form provided by the Journal.

## Conflicts of Interest

D.N., Y.Y., and G.Y. received personal fees from AstraZeneca. M.S. received personal fees from MSD K.K., AstraZeneca, Takeda Pharmaceutical, Bristol‐Myers Squibb, Daiichi Sankyo, and Chugai Pharmaceutical. K.Y. received personal fees from MSD K.K., AstraZeneca, Takeda Pharmaceutical, Chugai Pharmaceutical, Eli Lilly Japan, Boehringer‐Ingelheim, Bristol‐Myers Squibb, Nippon Kayaku, Merck Serono, and Pfizer. H.C. received personal fees from MSD K.K., AstraZeneca, Mochida Pharmaceutical, GlaxoSmithKline, Boehringer Ingelheim, Shionogi, Kyorin Pharmaceutical, Bayer, Sanofi, Daiichi Sankyo, Insmed, Taiho Pharmaceutical, Ono Pharmaceutical, and Chugai Pharmaceutical. All other authors have no conflicts of interest to declare.

## Data Availability

Data sharing is not applicable to this article as no datasets were generated or analysed during the current study.
